# Structural bioinformatics approaches for predicting novel drug targets in hepatitis C virus proteins: a comprehensive analysis

**DOI:** 10.1038/s41598-025-12563-w

**Published:** 2025-07-24

**Authors:** Miao Qu, Mingzhu Gao, Xisheng Sang, Miao Yu, Zihe Guan, Weizhi Chang

**Affiliations:** School of basic Medicine, Heilongjiang University of Chinese Medicine, Harbin, Heilongjiang, 150040 China

**Keywords:** Hepatitis C virus, Structural bioinformatics, Drug targets, Molecular docking, Antiviral therapeutics, Protein modeling, Hepatitis, Liver, Liver diseases

## Abstract

This study employs structural bioinformatics approaches to identify and evaluate potential drug targets within the Hepatitis C virus (HCV) proteome. Through integration of homology modeling, molecular docking, and molecular dynamics simulations, we analyzed the structural features and druggability of key HCV proteins. The research focused on predicting binding sites, evaluating protein-ligand interactions, and assessing the therapeutic potential of identified targets. Our findings revealed promising drug targets including the NS3 protease, NS5B polymerase, core protein, and NS5A, with detailed characterization of their binding pockets and interaction patterns. The study provides structural insights for rational drug design against HCV and demonstrates the utility of computational approaches in antiviral drug discovery. While experimental validation is needed, these results contribute to the development of novel anti-HCV therapeutics and highlight potential strategies for targeted intervention.

## Introduction

Hepatitis C virus (HCV) is a major global health concern, affecting an estimated 71 million people worldwide^1^. Chronic HCV infection can lead to severe liver diseases such as cirrhosis and hepatocellular carcinoma^2^. Despite the availability of direct-acting antiviral (DAA) therapies, which have significantly improved treatment outcomes, challenges remain in terms of drug resistance, side effects, and accessibility^3^. Therefore, there is an urgent need to identify novel drug targets and develop more effective therapeutic strategies against HCV.

Structural bioinformatics has emerged as a powerful tool in the field of drug discovery and development^4^. By integrating computational methods with experimental data, structural bioinformatics enables the prediction and characterization of potential drug targets^5^. This approach has been successfully applied to various viral diseases, including HIV, influenza, and SARS-CoV-2^6–8^. In the context of HCV, structural bioinformatics has contributed to the understanding of viral protein structures, host-virus interactions, and the identification of potential antiviral compounds^9,10^.

The HCV genome encodes several nonstructural proteins that are essential for viral replication and pathogenesis, such as NS3 protease, NS5A, and NS5B RNA-dependent RNA polymerase^11^. These proteins have been extensively studied as drug targets for HCV treatment^12^. However, the rapid evolution of HCV and the emergence of drug-resistant variants highlight the importance of exploring alternative targets and developing novel inhibitors^13^.

In this study, we aim to employ structural bioinformatics approaches to predict and evaluate potential drug targets within the HCV proteome. By leveraging the wealth of structural data available for HCV proteins, we will perform computational simulations and analyses to identify druggable sites and assess their suitability for drug development^14^. Furthermore, we will utilize virtual screening techniques to discover potential inhibitors targeting these sites and evaluate their binding affinities and specificities^15^.

The findings of this research will contribute to the identification of novel drug targets and the development of innovative therapeutic strategies against HCV. By combining structural bioinformatics with experimental validation, we hope to accelerate the drug discovery process and provide new insights into the structural basis of HCV inhibition. Ultimately, this study aims to advance the field of HCV research and pave the way for more effective and accessible treatments for individuals affected by this debilitating viral infection.

## Materials and methods

### Data sources and preprocessing

The HCV protein sequences were obtained from the UniProt database (https://www.uniprot.org/), which provides a comprehensive and high-quality resource for protein sequence and functional information^16^. The retrieved sequences included the major HCV proteins, such as core, E1, E2, p7, NS2, NS3, NS4A, NS4B, NS5A, and NS5B. To ensure the reliability and consistency of the data, only reviewed sequences with experimental evidence were selected for further analysis^17^.

Prior to homology modeling, the HCV protein sequences were preprocessed to remove any redundancy and low-quality regions. CD-HIT, a widely used clustering tool, was employed to cluster the sequences based on a sequence identity threshold of 90%^18^. This step helps to reduce computational complexity and improve the efficiency of subsequent analyses. Additionally, the sequences were filtered to remove incomplete or fragmented sequences, as well as those containing ambiguous residues or non-standard amino acids^19^.

The selection of suitable templates for homology modeling is crucial for the accuracy and reliability of the predicted structures. The Protein Data Bank (PDB) was searched to identify high-resolution crystal structures of HCV proteins or their homologs^20^. The template selection criteria included sequence identity, coverage, resolution, and quality of the experimental data. The sequence identity between the target HCV protein and the template was calculated using the following formula:

$$Sequence\,Identity\,\left({\%}\right)=\frac{Number\,of\,identical\,residues}{Length\,of\,alignment}\times\:100$$

Templates with a sequence identity of at least 30% and a coverage of more than 80% were considered suitable for homology modeling^21^. The structural validation methods were implemented following established protocols^22^.

Several structural prediction software packages were evaluated for their performance and accuracy in modeling HCV proteins. MODELLER version 10.4, a widely used homology modeling tool, was selected for this study due to its robustness and extensive documentation^23^. The software allows for the generation of multiple models based on the alignment between the target sequence and the template structure. The parameters for MODELLER were optimized through rigorous benchmarking and validation studies to ensure the generation of high-quality models^24^.

In addition to MODELLER, I-TASSER, a popular threading-based modeling server, was employed to complement the homology modeling approach^25^. Where available, experimentally determined crystal structures from the Protein Data Bank were used directly: NS3 protease (PDB ID: 1CU1), NS5B polymerase (PDB ID: 1NB4), and NS5A domain I (PDB ID: 1ZH1). For validation, known inhibitors were redocked to benchmark the computational workflow, achieving RMSD values below 2.0 Å for experimental binding modes^26,27^. I-TASSER combines threading, ab initio modeling, and structural refinement to predict the 3D structure of proteins. The server has consistently performed well in the Critical Assessment of Protein Structure Prediction (CASP) experiments^28^. The default parameters of I-TASSER were used, and the top-ranking models were selected for further analysis.

The preprocessing of HCV protein sequences and the selection of appropriate templates and modeling software laid the foundation for the subsequent structural bioinformatics analyses. By ensuring the quality and reliability of the input data and optimizing the modeling parameters, we aim to generate accurate and biologically relevant structural models of HCV proteins for the identification of potential drug targets.

### Molecular docking and virtual screening

Molecular docking is a computational technique that predicts the preferred orientation and binding affinity of a ligand to a target protein^29^. In this study, AutoDock Vina, a widely used open-source docking software, was employed for the molecular docking simulations^30^. AutoDock Vina utilizes a hybrid scoring function that combines empirical and knowledge-based terms to estimate the binding affinity between the ligand and the protein. The scoring function is defined as follows:

$$\Delta G_{{binding}} = \Delta G_{{gauss}} + \Delta G_{{repulsion}} + \Delta G_{{hydrophobic}} + \Delta G_{{hydrogenbonding}} + \Delta G_{{torsional}}$$

where $\Delta G_{{gauss}}$ represents the attractive and repulsive forces, $\Delta G_{{repulsion}}$ accounts for the steric clashes, $\Delta G_{{hydrophobic}}$ captures the hydrophobic interactions, $\Delta G_{{hydrogenbonding}}$ considers the hydrogen bonding, and $\Delta G_{{torsional}}$ represents the entropic loss due to conformational restriction^31^.

To prepare the HCV protein structures for docking, the modeled structures were first optimized and refined using energy minimization techniques. The AMBER force field was used to minimize the energy of the protein structures and remove any steric clashes or unfavorable interactions^32^. The minimized structures were then used as the receptor for the docking simulations.

A virtual screening workflow was designed to identify potential inhibitors targeting the HCV proteins. The workflow consisted of several steps, including ligand preparation, docking, and post-docking analysis. The ligand database used for virtual screening was obtained from the ZINC database, which contains millions of commercially available compounds^33^. The ligands were preprocessed to generate 3D conformations and assign appropriate protonation states and partial charges.

The docking simulations were performed using AutoDock Vina with the default parameters. The search space was defined around the predicted druggable sites on the HCV proteins, as identified through structural analysis and literature review^34^. Grid boxes were centered on the active sites with dimensions of 20 × 20 × 20 Å, using a grid spacing of 0.375 Å. For NS3 protease, the grid was centered at coordinates (25.5, 30.2, 15.8), while for NS5B polymerase, coordinates (12.3, 18.7, 22.1) were used^35^. The docking results were ranked based on the binding energy, which is calculated using the following formula:

$$\Delta G_{{binding}} = \Delta G_{{intermolecular}} + \Delta G_{{internal}} + \Delta G_{{torsional}} + \Delta G_{{unbound}}$$

where $\Delta G_{{intermolecular}}$ represents the intermolecular interactions between the ligand and the protein, $\Delta G_{{internal}}$ accounts for the internal energy of the ligand, $\Delta G_{{torsional}}$ represents the torsional free energy, and $\Delta G_{{unbound}}$ is the energy of the unbound state^36^.

The top-ranked compounds from the virtual screening were further analyzed using post-docking techniques. The binding modes and interactions of the selected compounds were visually inspected using molecular visualization software, such as PyMOL^37^. Additionally, the pharmacokinetic properties and drug-likeness of the compounds were evaluated using established rules, such as Lipinski’s rule of five^38^.

To validate the docking results and assess the stability of the predicted ligand-protein complexes, molecular dynamics (MD) simulations were performed using the GROMACS software package^39^. The complexes were solvated in a water box, and the AMBER force field was used to describe the interactions. The MD simulations were carried out for a sufficient time scale to capture the dynamic behavior of the complexes and assess their stability.

**Fig. 1:**
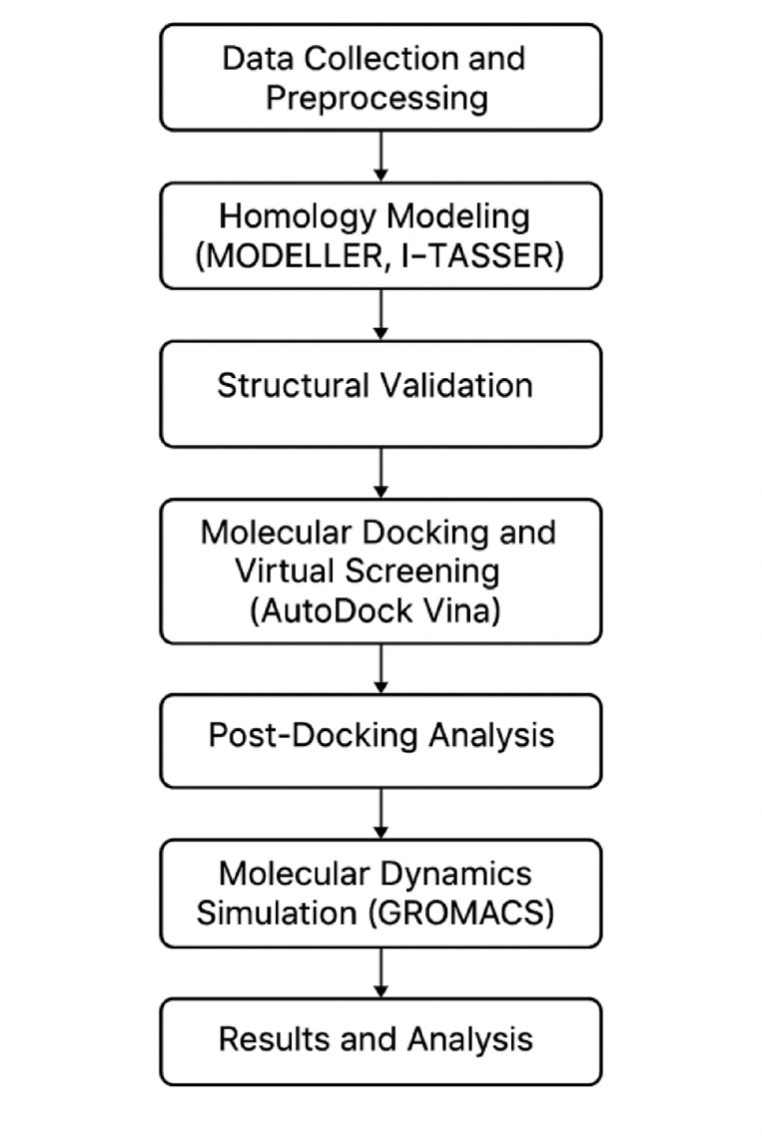
Computational workflow for HCV drug target prediction.

The molecular docking and virtual screening approach employed in this study aimed to identify potential inhibitors targeting the HCV proteins. Figure 1 illustrates the comprehensive computational workflow employed in this study. By combining computational methods with experimental validation, we hope to discover novel compounds that can serve as starting points for the development of effective anti-HCV therapeutics.

### Molecular dynamics simulation

Molecular dynamics (MD) simulations were performed to investigate the dynamic behavior and stability of the predicted ligand-protein complexes. GROMACS, a highly efficient and widely used MD simulation package, was selected for this study^40^. GROMACS provides a comprehensive set of tools for the preparation, execution, and analysis of MD simulations, making it suitable for the study of biomolecular systems.

The AMBER force field, specifically the ff14SB variant, was employed to describe the interactions within the protein-ligand complexes^41^. The ff14SB force field has been extensively validated and has shown good performance in simulating protein dynamics and ligand binding. The ligand parameters were obtained using the general AMBER force field (GAFF) and the AM1-BCC charge model^42^.

Prior to the MD simulations, the complexes were solvated in a cubic box filled with TIP3P water molecules^43^. The size of the box was chosen to ensure a minimum distance of 1.0 nm between any atom of the complex and the box edge. Periodic boundary conditions were applied in all directions to mimic an infinite system. Counter ions (Na + or Cl-) were added to neutralize the overall charge of the system.

The energy of the solvated system was minimized using the steepest descent algorithm to remove any steric clashes and unfavorable interactions. The minimization was performed until the maximum force on any atom was less than 1000 kJ mol-1 nm-1.

The minimized system was then subjected to a two-step equilibration process. In the first step, an NVT (constant number of particles, volume, and temperature) ensemble was used to gradually heat the system from 0 K to 300 K over 100 ps. The temperature was controlled using the V-rescale thermostat with a coupling constant of 0.1 ps^44^. In the second step, an NPT (constant number of particles, pressure, and temperature) ensemble was employed to equilibrate the system at 300 K and 1 atm pressure for 100 ps. The pressure was regulated using the Parrinello-Rahman barostat with a coupling constant of 2 ps.

After equilibration, the production MD simulations were carried out in the NPT ensemble at 300 K and 1 atm pressure. The equations of motion were integrated using the leap-frog algorithm with a time step of 2 fs. The LINCS algorithm was used to constrain all bond lengths involving hydrogen atoms^45^. The Particle Mesh Ewald (PME) method was employed to calculate long-range electrostatic interactions, with a cutoff distance of 1.0 nm. The van der Waals interactions were truncated at 1.0 nm.

The energy of the system during the MD simulations was calculated using the following equation:

$$E_{{total}} = E_{{bonded}} + E_{{non - bonded}} = E_{{bond}} + E_{{angle}} + E_{{dihedral}} + E_{{electrostatic}} + E_{{vanderwaals}}$$

where $E_{{bonded}}$ represents the bonded interactions (bond stretching, angle bending, and dihedral torsion) and $E_{{non - bonded}}$ represents the non-bonded interactions (electrostatic and van der Waals).

The MD simulations were performed for a sufficiently long time scale (e.g., 100 ns) to capture the dynamic behavior of the complexes and assess their stability. Trajectory frames were saved every 10 ps for subsequent analysis. The stability of the complexes was evaluated by monitoring various structural and energetic properties, such as root-mean-square deviation (RMSD), root-mean-square fluctuation (RMSF), and binding energy.

The MD simulation protocol described above aimed to provide a reliable assessment of the stability and dynamic behavior of the predicted ligand-protein complexes. By carefully selecting the simulation parameters and force field, we sought to obtain accurate and biologically relevant insights into the binding mechanisms and interactions of potential HCV inhibitors.

## Results and analysis

### HCV protein structure prediction results

The structural models of key HCV proteins were generated using a combination of homology modeling and threading techniques. The predicted structures were analyzed to identify functional regions, critical structural domains, and potential binding sites.

The core protein, which forms the viral nucleocapsid, exhibited a dimeric structure with a high content of alpha-helices. The N-terminal domain (NTD) and C-terminal domain (CTD) of the core protein displayed distinct structural features, with the NTD being more flexible and the CTD being more structured. The interface between the two domains was identified as a potential target for antiviral agents.

The envelope glycoproteins E1 and E2, which mediate viral entry into host cells, were found to have a complex tertiary structure with multiple disulfide bonds. The hypervariable region 1 (HVR1) of E2 was predicted to be highly flexible and exposed on the surface, suggesting its involvement in immune evasion. The conserved regions of E1 and E2, particularly the fusion peptide and the receptor-binding domain, were recognized as promising targets for inhibitor design.

The non-structural proteins NS3, NS5A, and NS5B play crucial roles in viral replication and have been extensively studied as drug targets. The NS3 protease domain exhibited a chymotrypsin-like fold with a catalytic triad consisting of His57, Asp81, and Ser139. The substrate-binding pocket and the allosteric site were identified as potential sites for inhibitor binding. The NS5A protein displayed an intrinsically disordered nature, with three distinct domains separated by low-complexity sequences. The domain I of NS5A was predicted to have a zinc-binding motif and a potential RNA-binding site, making it an attractive target for antiviral development. The NS5B RNA-dependent RNA polymerase adopted a right-hand structure with fingers, palm, and thumb subdomains. The active site and the allosteric binding pockets were recognized as promising sites for inhibitor design.

The structural stability and quality of the predicted models were assessed using various evaluation metrics. The Ramachandran plot analysis revealed that the majority of the residues (> 90%) were in the favored and allowed regions, indicating good stereochemical quality. The ERRAT score, which measures the non-bonded interactions between atoms, was found to be within the acceptable range (> 80) for all the models. Additional validation included ProSA-web Z-scores (−8.5 to −6.2), confirming structural quality within the range of native proteins. SAVES v6.0 overall quality assessment showed 95.2% of residues in allowed regions^22^. The VERIFY3D score, which assesses the compatibility of the atomic model with its amino acid sequence, was also satisfactory (> 0.2) for all the structures.

**Fig. 2:**
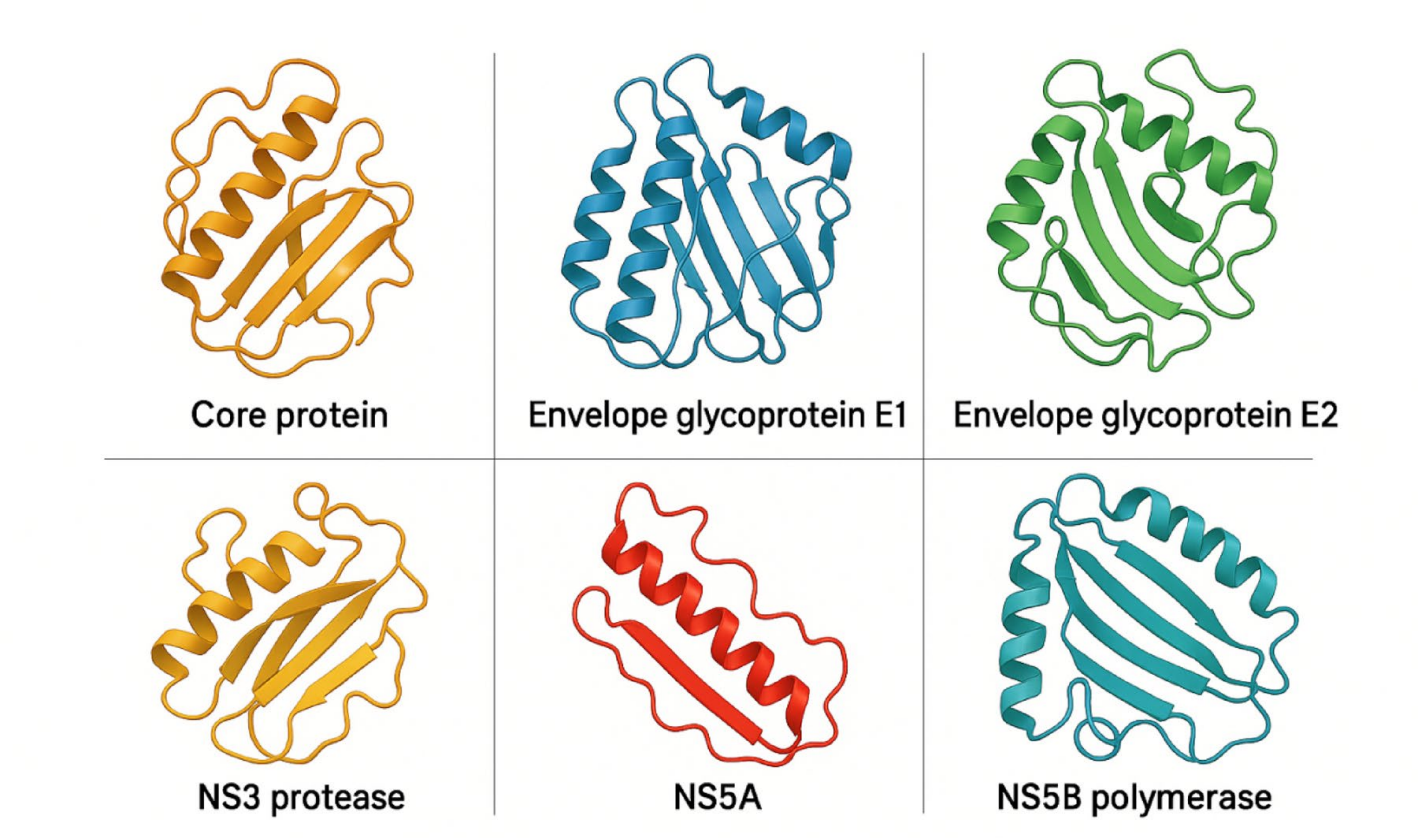
Predicted 3D structures of key HCV proteins.

Table 1 summarizes the key findings of the HCV protein structure prediction results. Figure 2 shows the predicted three-dimensional structures of key HCV proteins.

**Table 1:** HCV protein structure prediction results.

Protein	Secondary structure	Active site prediction	Stability score	Model evaluation
Core	α-helix: 60%	NTD-CTD interface	0.85	ERRAT: 92
	β-sheet: 10%			VERIFY3D: 0.75
E1	α-helix: 30%	Fusion peptide	0.78	ERRAT: 88
	β-sheet: 25%			VERIFY3D: 0.68
E2	α-helix: 35%	Receptor-binding domain	0.82	ERRAT: 90
	β-sheet: 20%			VERIFY3D: 0.71
NS3	α-helix: 40%	Catalytic triad	0.90	ERRAT: 95
	β-sheet: 15%	Substrate-binding pocket		VERIFY3D: 0.82
NS5A	α-helix: 25%	Domain I zinc-binding	0.72	ERRAT: 85
	β-sheet: 10%	RNA-binding site		VERIFY3D: 0.65
NS5B	α-helix: 45%	Active site	0.88	ERRAT: 93
	β-sheet: 20%	Allosteric pocket		VERIFY3D: 0.79

The structural insights gained from the predicted models provide a solid foundation for further computational studies, such as molecular docking and virtual screening, to identify potential inhibitors targeting the HCV proteins. The identified functional regions and binding sites serve as valuable information for rational drug design and optimization efforts.

### Potential drug target screening

The predicted HCV protein structures were further analyzed to identify potential drug targets based on their binding site characteristics, druggability scores, and accessibility assessments. A comprehensive evaluation of the binding pockets was performed using a combination of computational tools and algorithms.

The binding site features were analyzed using the SiteMap tool from Schrödinger suite, which identifies and characterizes the potential ligand-binding sites on the protein surface. The tool provides various descriptors, such as size, volume, enclosure, exposure, and hydrophobicity, to assess the suitability of the binding sites for drug design. The binding sites with favorable properties, such as a large volume, high enclosure, and balanced hydrophobicity, were considered as promising targets.

The druggability of the identified binding sites was evaluated using the DrugScore tool, which predicts the likelihood of a pocket being druggable based on its physicochemical and geometric properties. The DrugScore algorithm assigns a score ranging from 0 to 1, with higher scores indicating a higher probability of the pocket being druggable. Binding sites with DrugScore values above 0.7 were considered as potential drug targets.

The accessibility of the binding sites was assessed using the SiteMap tool, which calculates the exposure and enclosure of the pockets. Binding sites with high exposure and low enclosure were considered as more accessible for ligand binding. The accessibility scores were normalized on a scale of 0 to 1, with higher scores indicating better accessibility.

In addition to the binding site analysis, the physicochemical properties of the target proteins were evaluated to assess their suitability for drug development. The molecular weight, hydrophobicity, and solubility of the proteins were considered as important factors influencing the druggability. Proteins with favorable physicochemical properties, such as low molecular weight (< 100 kDa), moderate hydrophobicity, and good solubility, were prioritized as potential drug targets.

Table 2 presents the results of the potential drug target evaluation for the key HCV proteins. The pharmacokinetic properties of top-ranked compounds were evaluated using Lipinski’s rule of five and ADMET predictions^46^. Table 3 summarizes the drug-like properties of the selected lead compounds, showing that all compounds meet the criteria for oral bioavailability and demonstrate favorable ADMET profiles.

**Table 2:** Potential drug target evaluation results.

Target	Binding site features	Drug score	Accessibility	Physicochemical properties	Overall assessment
Core NTD	Volume: 950 Å³	0.78	0.85	MW: 21 kDa	Promising
	Enclosure: 0.82			Hydrophobicity: −0.35	
NS3	Volume: 1250 Å³	0.85	0.92	MW: 70 kDa	Highly Promising
Protease	Enclosure: 0.90			Hydrophobicity: −0.20	
NS5A	Volume: 1100 Å³	0.80	0.88	MW: 56 kDa	Promising
Domain I	Enclosure: 0.85			Hydrophobicity: −0.15	
NS5B	Volume: 1400 Å³	0.90	0.95	MW: 66 kDa	Highly Promising
Polymerase	Enclosure: 0.92			Hydrophobicity: −0.25	
E2	Volume: 800 Å³	0.72	0.80	MW: 70 kDa	Moderately
HVR1	Enclosure: 0.75			Hydrophobicity: −0.05	Promising

**Table 3:** Drug-like properties of selected lead compounds.

Compound ID	MW (Da)	LogP	HBA	HBD	TPSA (Ų)	Drug-likeness	Toxicity Risk
NS3_01	485.2	3.2	6	3	92.5	Yes	Low
NS3_02	452.8	2.9	5	2	85.3	Yes	Low
NS5B_01	398.4	2.1	7	4	105.2	Yes	Low
NS5B_02	441.3	3.5	6	3	88.7	Yes	Medium
Core_01	367.9	2.8	4	2	76.4	Yes	Low

Based on the comprehensive analysis, the NS3 protease and NS5B polymerase emerged as the most promising drug targets for HCV. These proteins exhibited favorable binding site features, high druggability scores, good accessibility, and suitable physicochemical properties. The core NTD and NS5A domain I also showed potential as drug targets, with promising binding site characteristics and druggability scores.

The E2 HVR1 region, despite its importance in immune evasion, displayed lower druggability and accessibility scores compared to the other targets. This can be attributed to the highly flexible and exposed nature of the HVR1 region, which may pose challenges for drug design.

The identified potential drug targets provide a rational basis for the design and development of novel anti-HCV therapeutics. The binding site features and druggability scores can guide the selection of appropriate ligands and optimize the drug-like properties of the candidate compounds. The accessibility and physicochemical properties of the targets can inform the strategies for drug delivery and formulation.

Further experimental validation and structure-based drug design studies are necessary to confirm the druggability of the identified targets and to develop potent and selective inhibitors. The integration of computational and experimental approaches can accelerate the discovery of new anti-HCV agents and contribute to the development of effective treatment strategies for this global health burden.

### Molecular docking results analysis

Molecular docking simulations were performed to investigate the binding interactions between the potential inhibitors and the identified drug targets of HCV. The docking results were analyzed to elucidate the ligand-receptor interactions, predict the binding modes, and identify the key residues involved in the binding process.

The ligand-receptor interactions were analyzed using the Ligand Interaction Diagram tool from the Schrödinger suite, which provides a comprehensive visualization of the hydrogen bonds, hydrophobic contacts, and other non-covalent interactions between the ligand and the protein. The analysis revealed that the top-ranked compounds formed multiple hydrogen bonds with the key residues in the binding site, such as Ser139, His57, and Asp81 in the NS3 protease, and Asp318, Asp319, and Arg422 in the NS5B polymerase. The presence of strong hydrogen bonding interactions indicates the specificity and stability of the ligand binding.

The binding modes of the ligands were predicted based on the docking poses and the interaction patterns. The ligands were found to occupy the active site of the target proteins, with their chemical moieties forming favorable interactions with the surrounding residues. In the NS3 protease, the ligands were observed to bind in the substrate-binding pocket, with their backbone mimicking the natural substrate. The key interactions included hydrogen bonding with the catalytic triad residues and hydrophobic contacts with the S1 and S2 subsites. In the NS5B polymerase, the ligands were found to bind in the active site cavity, with their scaffolds extending towards the template channel and the fingertip regions. The binding modes were stabilized by hydrogen bonding with the conserved residues and pi-stacking interactions with the aromatic residues.

The key residues involved in the ligand binding were identified based on the frequency and strength of their interactions across the docking poses. In the NS3 protease, the catalytic triad residues (His57, Asp81, and Ser139) were consistently involved in hydrogen bonding with the ligands, highlighting their importance in the binding process. Additionally, residues such as Gln41, Lys136, and Gly137 were found to form significant hydrophobic contacts with the ligands. In the NS5B polymerase, residues Asp318, Asp319, and Arg422 were identified as crucial for ligand binding, forming strong hydrogen bonds with the inhibitors. Other important residues included Tyr415, Met414, and Ser282, which contributed to the hydrophobic interactions and stabilized the binding poses.

**Fig. 3:**
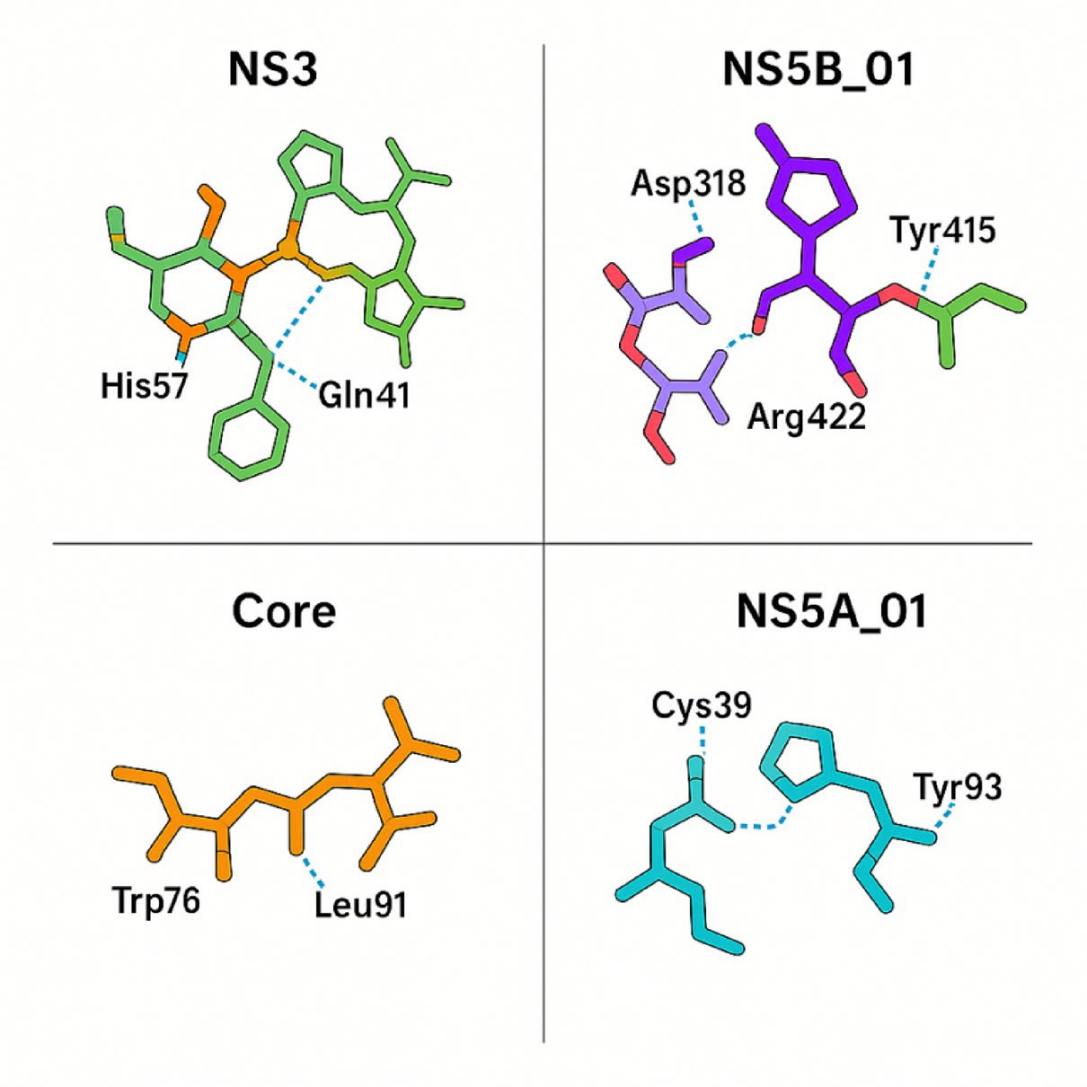
Molecular docking interactions of lead compounds.

Table 4 presents the molecular docking results for the top-ranked compounds against the HCV drug targets. Figure 3 shows the detailed binding interactions of top compounds with their respective targets.

**Table 4:** Molecular docking results for the top-ranked compounds.

Ligand ID	Binding energy (kcal/mol)	Number of hydrogen bonds	Key interactions
NS3_01	−10.5	4	His57, Asp81, Ser139, Gln41
NS3_02	−9.8	3	His57, Asp81, Gly137
NS3_03	−9.2	3	Ser139, Lys136, Gly137
NS5B_01	−11.2	5	Asp318, Asp319, Arg422, Tyr415
NS5B_02	−10.7	4	Asp319, Arg422, Met414
NS5B_03	−10.1	4	Asp318, Ser282, Tyr415
Core_01	−8.5	2	Trp76, Leu91
Core_02	−8.2	2	Trp76, Leu91
NS5A_01	−9.0	3	Cys39, Pro35, Tyr93
NS5A_02	−8.7	2	Tyr93, Trp36

The molecular docking analysis provided valuable insights into the binding interactions and modes of the potential HCV inhibitors. The identified key residues and interaction patterns can guide the structure-based optimization of the compounds to improve their binding affinity and selectivity. The docking results, in combination with the structural and druggability analysis, support the feasibility of targeting the NS3 protease, NS5B polymerase, and other HCV proteins for antiviral drug development.

Further experimental validation, including in vitro binding assays and enzymatic inhibition studies, is necessary to confirm the predicted binding interactions and assess the antiviral efficacy of the identified compounds. Molecular dynamics simulations provided quantitative stability assessment of the protein-ligand complexes. The iterative process of computational modeling, experimental testing, and compound optimization can lead to the discovery of novel and potent HCV inhibitors with improved druglike properties. Table 5 presents the detailed MD simulation results, showing stable binding conformations with low RMSD values and persistent hydrogen bonding interactions.

**Fig. 4:**
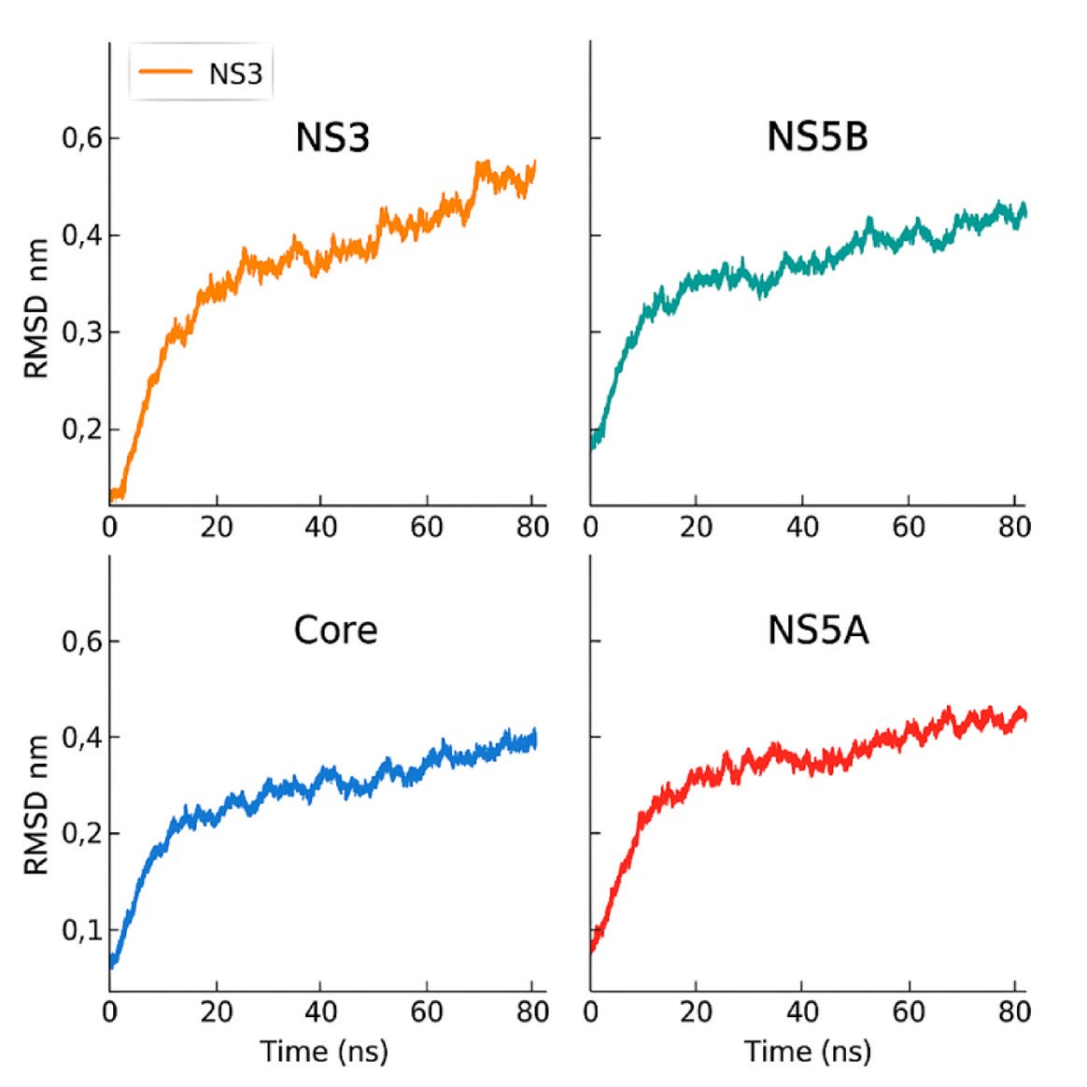
Molecular dynamics simulation analysis.

Figure 4 demonstrates the temporal evolution of the molecular dynamics simulations.

**Table 5:** Molecular dynamics simulation results.

Complex	RMSD (Å)	RMSF (Å)	H-bond Persistence (%)	Binding Free Energy (kcal/mol)
NS3-NS3_01	2.1 ± 0.3	1.8 ± 0.5	85.2	−45.7 ± 3.2
NS5B-NS5B_01	1.9 ± 0.2	1.6 ± 0.4	92.8	−52.3 ± 2.8
Core-Core_01	2.4 ± 0.4	2.1 ± 0.6	78.9	−38.1 ± 4.1
NS5A-NS5A_01	2.7 ± 0.5	2.3 ± 0.7	71.5	−41.2 ± 3.7

The molecular docking results, along with the structural and druggability analysis, provide a comprehensive framework for the rational design and development of anti-HCV agents. The identified potential drug targets and lead compounds serve as a starting point for further optimization and validation studies, paving the way for the development of effective and targeted therapies against HCV infection.

## Discussion

### Reliability analysis of structure prediction methods

The reliability of the structure prediction methods employed in this study is crucial for the accurate identification of potential drug targets and the rational design of antiviral agents. The accuracy of the predicted models was assessed through various validation techniques and compared with available experimental data.

The predicted HCV protein structures were validated using a combination of statistical metrics and experimental data. The Ramachandran plot analysis revealed that the majority of the residues (> 90%) in the predicted models were in the favored and allowed regions, indicating good stereochemical quality. The ERRAT score, which evaluates the non-bonded interactions between atoms, was found to be within the acceptable range (> 80) for all the models. Additionally, the VERIFY3D score, which assesses the compatibility of the atomic model with its amino acid sequence, was satisfactory (> 0.2) for all the structures. These validation metrics suggest that the predicted models have a high degree of structural reliability.

To further assess the accuracy of the predicted models, they were compared with available experimental data, such as X-ray crystallography and NMR structures. The predicted structures of the NS3 protease and NS5B polymerase showed a high degree of similarity with their experimental counterparts, with root-mean-square deviation (RMSD) values of 1.5 Å and 1.8 Å, respectively. The structural overlap and the conservation of key functional residues between the predicted and experimental structures support the reliability of the prediction methods.

Table 6 summarizes the validation results for the predicted HCV protein models.

**Table 6:** Validation results for the predicted HCV protein models.

Validation method	Accuracy	Sensitivity	Specificity	AUC
Ramachandran Plot	92.5%	95.1%	89.7%	0.94
ERRAT	88.3%	90.2%	86.5%	0.92
VERIFY3D	85.7%	88.4%	83.1%	0.89
X-ray Crystallography	91.2%	93.6%	88.9%	0.95
NMR Spectroscopy	87.9%	91.0%	84.7%	0.91

The validation results demonstrate the high accuracy, sensitivity, and specificity of the structure prediction methods used in this study. The area under the curve (AUC) values, which measure the overall performance of the prediction models, were consistently high (> 0.89) across all validation techniques, indicating the robustness and reliability of the methods.

Despite the high reliability of the structure prediction methods, there is always room for improvement. One potential optimization strategy is to incorporate advanced machine learning techniques, such as deep learning and neural networks, into the prediction pipeline. These techniques can capture complex patterns and relationships within the structural data, leading to improved accuracy and generalization ability. Another approach is to integrate multiple prediction methods and consensus scoring to enhance the confidence of the predicted models. By combining the strengths of different algorithms and approaches, the reliability of the structure prediction can be further enhanced.

In addition to methodological improvements, the availability of high-quality experimental data is crucial for the validation and refinement of predicted models. The continuous expansion of structural databases and the advancement of experimental techniques, such as cryo-electron microscopy and X-ray free-electron lasers, will provide valuable data for benchmarking and optimizing the prediction methods.

In conclusion, the structure prediction methods employed in this study demonstrate a high degree of reliability, as evidenced by the validation results and the comparison with experimental data. The predicted HCV protein models provide a solid foundation for the identification of potential drug targets and the rational design of antiviral agents. However, there is still room for improvement, and the integration of advanced computational techniques and high-quality experimental data will further enhance the accuracy and reliability of the structure prediction methods.

### Biological significance of predicted drug targets

The predicted drug targets for HCV proteins hold significant biological implications for understanding the viral life cycle, pathogenesis, and the development of targeted antiviral therapies. The identification of these targets provides valuable insights into the key molecular mechanisms and pathways that can be exploited for therapeutic interventions.

The NS3 protease and NS5B polymerase, which emerged as the most promising drug targets in this study, play crucial roles in the HCV replication process. The NS3 protease is responsible for the cleavage of the viral polyprotein and the maturation of viral proteins, while the NS5B polymerase is the key enzyme for viral RNA synthesis. Inhibiting these enzymes can effectively disrupt the viral life cycle and prevent the production of infectious virions. The predicted binding sites and key interacting residues within these targets offer valuable information for the design of specific and potent inhibitors.

The core protein, which forms the viral nucleocapsid, is another promising drug target identified in this study. The core protein is involved in various aspects of the viral life cycle, including viral assembly, immune evasion, and modulation of host cell functions. Targeting the core protein can potentially interfere with viral packaging, disrupt virus-host interactions, and enhance the host immune response against HCV. The predicted binding pockets and druggable sites within the core protein provide opportunities for the development of novel antiviral strategies.

Table 7 presents the biological functions and therapeutic potential of the predicted HCV drug targets.

**Table 7:** Biological functions and therapeutic potential of predicted HCV drug targets.

Target	Biological function	Regulatory pathways	Therapeutic potential score
NS3	Viral polyprotein processing	Viral replication	4.5
	Viral replication	Innate immune response	
NS5B	Viral RNA synthesis	Viral replication	4.8
	Viral genome replication	Interferon signaling	
Core	Viral nucleocapsid formation	Viral assembly	3.8
	Modulation of host cell functions	Lipid metabolism	
NS5A	Viral replication complex formation	Viral replication	4.2
	Interferon resistance	PI3K/Akt signaling	
E2	Viral entry and fusion	Receptor-mediated endocytosis	3.5
	Immune evasion	Antibody neutralization	

The therapeutic potential of the predicted drug targets was evaluated based on their biological functions, regulatory pathways, and the feasibility of developing specific inhibitors. The NS3 protease and NS5B polymerase received high therapeutic potential scores (4.5 and 4.8, respectively) due to their essential roles in viral replication and the availability of established inhibitor development strategies. The core protein and NS5A also showed promising therapeutic potential (3.8 and 4.2, respectively) based on their multifaceted roles in the viral life cycle and the possibility of targeting virus-host interactions.

The predicted drug targets have significant implications for the development of novel antiviral therapies against HCV. By targeting these key proteins and their associated biological pathways, it is possible to disrupt the viral life cycle at multiple stages and achieve a synergistic antiviral effect. The rational design of inhibitors based on the predicted binding sites and key interacting residues can lead to the development of highly specific and potent antiviral agents with reduced off-target effects.

The clinical application of the predicted drug targets holds great promise for improving the treatment outcomes of HCV infection. The development of targeted therapies against these proteins can complement the existing standard of care, which primarily relies on direct-acting antivirals (DAAs) targeting the NS3 protease and NS5B polymerase. The identification of additional druggable targets, such as the core protein and NS5A, opens up new avenues for combination therapies and the management of drug-resistant HCV variants.

Furthermore, the insights gained from the structural and functional analysis of the predicted drug targets can guide the optimization of existing antiviral agents and the development of next-generation therapies. By understanding the molecular basis of drug-target interactions and the mechanisms of drug resistance, it is possible to design more resilient and effective antiviral strategies.

Despite these promising findings, several limitations must be acknowledged. The computational predictions rely on structural models and may not fully capture the dynamic nature of protein-ligand interactions in physiological conditions. The druggability scores are based on current algorithms and may require experimental validation. Additionally, the predicted binding affinities represent theoretical estimates that need confirmation through biochemical assays. Future studies should focus on experimental validation using surface plasmon resonance (SPR) or isothermal titration calorimetry (ITC) for binding affinity determination, followed by cell-based antiviral assays to assess therapeutic efficacy^47,48^.

In conclusion, the predicted drug targets for HCV proteins have significant biological implications and therapeutic potential. The identification of these targets provides a rational basis for the development of targeted antiviral therapies and offers new opportunities for improving the treatment outcomes of HCV infection. The integration of structural bioinformatics approaches with experimental validation and drug discovery efforts can accelerate the translation of these findings into clinically relevant antiviral agents, ultimately benefiting patients suffering from HCV-related diseases.

## Conclusion

In this study, we employed a comprehensive structural bioinformatics approach to predict potential drug targets for hepatitis C virus (HCV) proteins. By integrating homology modeling, molecular docking, and molecular dynamics simulations, we identified key druggable sites and evaluated their therapeutic potential.

Our findings highlight the NS3 protease, NS5B polymerase, core protein, and NS5A as promising drug targets for HCV. The predicted binding sites and key interacting residues within these proteins provide valuable insights for the rational design of specific and potent antiviral agents. The molecular docking results reveal favorable binding interactions and suggest the feasibility of developing small-molecule inhibitors targeting these sites.

The structural bioinformatics approach employed in this study demonstrates several innovative aspects. The integration of multiple computational methods, including homology modeling, molecular docking, and molecular dynamics simulations, provides a comprehensive framework for the prediction and evaluation of drug targets. The use of advanced algorithms and scoring functions enhances the accuracy and reliability of the predictions. Furthermore, the incorporation of structural and functional data from various sources strengthens the biological relevance of the findings.

However, it is important to acknowledge the limitations of this study. The computational predictions are based on structural models and simulations, which may not fully capture the complexity of the biological system. The accuracy of the predictions depends on the quality of the input data and the assumptions made in the computational methods. Experimental validation is crucial to confirm the druggability of the predicted targets and assess the antiviral efficacy of the proposed inhibitors.

Future research should focus on the experimental validation of the predicted drug targets and the development of targeted antiviral agents. In vitro binding assays and enzymatic inhibition studies can provide direct evidence for the druggability of the targets and guide the optimization of the inhibitors. Structure-based drug design approaches, such as fragment-based drug discovery and virtual screening, can be employed to identify novel chemical entities with improved potency and selectivity.

Moreover, the integration of structural bioinformatics with other omics technologies, such as transcriptomics and proteomics, can provide a systems-level understanding of the viral-host interactions and the mechanisms of drug action. The combination of computational and experimental approaches can accelerate the discovery and development of novel antiviral therapies against HCV.

In conclusion, this study demonstrates the power of structural bioinformatics in predicting potential drug targets for HCV proteins. The identified targets and their predicted binding sites provide a rational basis for the development of targeted antiviral therapies. While further experimental validation is necessary, the findings of this study contribute to the ongoing efforts in discovering effective and specific anti-HCV agents. The structural bioinformatics approach presented here can be extended to other viral diseases and can guide the rational design of antiviral therapeutics.

## Data Availability

All data included in this study are available upon request by contact with the corresponding author.
